# Potential clinical impact of cardiovascular magnetic resonance assessment of ejection fraction on eligibility for cardioverter defibrillator implantation

**DOI:** 10.1186/1532-429X-14-69

**Published:** 2012-10-08

**Authors:** Subodh B Joshi, Kim A Connelly, Laura Jimenez-Juan, Mark Hansen, Anish Kirpalani, Paul Dorian, Iqwal Mangat, Abdul Al-Hesayen, Andrew M Crean, Graham A Wright, Andrew T Yan, Howard Leong-Poi

**Affiliations:** 1 Keenan Research Centre at the Li Ka Shing Knowledge Institute, Division of Cardiology, St Michael’s Hospital, University of Toronto, 30 Bond St, Toronto, ON, M5B 1W8, Canada; 2 Department of Medical Imaging, University Health Network, University of Toronto, Toronto, Canada; 3 Division of Cardiology, Schulich Heart Program, Sunnybrook Health Sciences Centre, Toronto, Canada; 4 Department of Medical Imaging, St Michael’s Hospital, University of Toronto, Toronto, Canada; 5 Division of Cardiology, University Health Network, University of Toronto, Toronto, Canada; 6 Department of Medical Biophysics, University of Toronto and Schulich Heart Program, Sunnybrook Health Sciences Centre, Toronto, Canada

**Keywords:** Implantable cardioverter defibrillators, Ejection Fraction, Echocardiography, Cardiovascular Magnetic Resonance

## Abstract

**Background:**

For the primary prevention of sudden cardiac death, guidelines provide left ventricular ejection fraction (EF) criteria for implantable cardioverter defibrillator (ICD) placement without specifying the technique by which it should be measured. We sought to investigate the potential impact of performing cardiovascular magnetic resonance (CMR) for EF on ICD eligibility.

**Methods:**

The study population consisted of patients being considered for ICD implantation who were referred for EF assessment by CMR. Patients who underwent CMR within 30 days of echocardiography were included. Echocardiographic EF was determined by Simpson’s biplane method and CMR EF was measured by Simpson’s summation of discs method.

**Results:**

Fifty-two patients (age 62±15 years, 81% male) had a mean EF of 38 ± 14% by echocardiography and 35 ± 14% by CMR. CMR had greater reproducibility than echocardiography for both intra-observer (ICC, 0.98 vs 0.94) and inter-observer comparisons (ICC 0.99 vs 0.93). The limits of agreement comparing CMR and echocardiographic EF were – 16 to +10 percentage points. CMR resulted in 11 of 52 (21%) and 5 of 52 (10%) of patients being reclassified regarding ICD eligibility at the EF thresholds of 35 and 30% respectively. Among patients with an echocardiographic EF of between 25 and 40%, 9 of 22 (41%) were reclassified by CMR at either the 35 or 30% threshold. Echocardiography identified only 1 of the 6 patients with left ventricular thrombus noted incidentally on CMR.

**Conclusions:**

CMR resulted in 21% of patients being reclassified regarding ICD eligibility when strict EF criteria were used. In addition, CMR detected unexpected left ventricular thrombus in almost 10% of patients. Our findings suggest that the use of CMR for EF assessment may have a substantial impact on management in patients being considered for ICD implantation.

## Background

Implantable cardioverter defibrillator (ICD) placement has been shown to reduce the risk of sudden cardiac death in patients with severe left ventricular dysfunction [1-3]. Guidelines recommend ICD implantation for primary prevention in patients with left ventricular ejection fraction (EF) below 30% or 35% depending on etiology and symptoms [1-3]. EF is the best validated of the predictors of sudden death and is the main variable used in clinical practice to make treatment decisions about ICD eligibility. While specific EF criteria are suggested, the technique by which EF should be measured is not specified in the guidelines [1-3]. In clinical practice, echocardiography is the modality most commonly used to assess left ventricular function and was one of several modalities used in trials establishing the efficacy of ICD therapy [4-6]. However, cardiovascular magnetic resonance (CMR) is now considered the gold standard for EF measurement and is known to have greater reproducibility than echocardiography [7-9]. When strict EF thresholds are used for primary prevention ICD placement, it is unknown whether differences between modalities would alter clinical decisions. We sought to investigate the potential clinical impact of performing CMR for EF in patients being evaluated for ICD therapy.

## Methods

The study population consisted of patients being considered for ICD implantation at two academic medical centres between March 20, 2007 and Nov 19, 2010. Patients referred for CMR with the indications of both “ICD” and “EF” assessment were identified from a database. All patients who had undergone CMR within 30 days of echocardiography were included in the study. No patients were excluded based on image quality, to reflect a “real world” population of patients. Two patients were in atrial fibrillation, and both were well rate-controlled resulting in satisfactory image quality for both echocardiographic and CMR examinations. Patients undergoing coronary revascularization or cardiac resynchronization therapy between echocardiography and CMR examinations were excluded. Clinical records were reviewed to identify risk factors and co-morbid clinical conditions. Ischemic heart disease was determined to be the cause of left ventricular dysfunction based on a history of myocardial infarction, coronary revascularisation or a stenosis of greater than 70% severity in at least one major epicardial coronary artery. Approval was obtained from the institutional research ethics boards.

### Echocardiography

A commercially available clinical system (IE 33, Philips Healthcare Canada) was used to perform standard clinical two-dimensional echocardiography. All studies included focused imaging of the left ventricle in the apical two and four chamber views for EF assessment.

Echocardiographic studies were read by expert observers (H.L.P., M.H.) on commercially available software (Xcelera, Philips Healthcare Canada). Observers performed their analyses independent of one another and were blinded to CMR results. All studies were read a second time in random order after a 4 week interval. EF was measured by Simpson’s biplane method by manual tracing of endocardial borders on apical 2 and 4 chamber images according to American Society of Echocardiography Guidelines [10]. A qualitative assessment was also made regarding the pattern of left ventricular dysfunction; marked variability in function from one segment to another suggestive of an ischemic cause was considered a regional pattern, while a uniform pattern of left ventricular dysfunction was deemed global. The presence or absence of thrombus was assessed using all images available in the echocardiographic study. Echocardiographic contrast agent for left ventricular opacification was not used.

### Cardiovascular Magnetic Resonance (CMR)

CMR was performed on one of two commercially available 1.5 Telsa field strength systems (Achieva, Philips Medical Systems, Best, Netherlands and Signa Excite, GE Medical Systems Milwaukee, WI). A standard clinical CMR was performed including a segmented k-space cine steady state free precession (SSFP) series in a left ventricular short axis orientation. Slice thickness was 8 mm with no gap and the in-plane resolution was approximately 1.6 x 1.6 mm. There were 20 – 25 phases per cardiac cycle resulting in a temporal resolution of <50 ms. Late gadolinium enhancement images were acquired approximately 10 minutes after intravenous administration of 0.1-0.2 mmol/kg of gadolinium (gadobutrol or gadopentate dimeglumine) with the inversion time adjusted to optimally null the normal myocardium [11].

CMR images were analyzed by experienced readers (L.J., S.J.), blinded to the other’s results, on a single commercially available platform (CMR42, Circle Cardiovascular Imaging, Calgary, Canada). EF was measured by manual planimetry of the left ventricular endocardium in short axis cine images at end-systole and end-diastole. End-diastolic and end-systolic phases were chosen independently by each observer based on maximum and minimum volume, with multiple phases contoured in case of doubt. At the basal slice, the ventricular cavity was differentiated from the atrium by the presence of ventricular myocardium and was confirmed on a co-registered long-axis image. Papillary muscles were excluded from the left ventricular mass, that is, they were ignored and included in the left ventricular cavity volume. Both cine SSFP and late gadolinium enhancement images were used to assess for left ventricular thrombus. The diagnosis of thrombus was made based upon the identification of low signal intensity filling defects within the left ventricular cavity adjacent to myocardium with severely impaired function [12,13]. Thrombus was distinguished from microvascular obstruction related to infarction by its location within the left ventricular cavity (as opposed to the myocardium) and its stability of size on consecutive late enhancement images [14]. In case of disagreement another expert reader independently adjudicated the results (K.C., A.Y.).

### Statistical analysis

Statistical analyses were performed on Stata version 10 (Statacorp, College Station, Texas, USA) and MedCalc version 11.6.1.0 (MedCalc Software, Mariakerke, Belgium). Continuous variables were expressed as means and standard deviations, or medians and inter-quartile ranges (IQR) for data that were not normally distributed. Methods of EF assessment were compared using Bland-Altman analysis, Student’s paired t-test and Pearson’s correlation coefficient. Reproducibility was also assessed using the intra-class correlation coefficient (ICC). The Kappa statistic was used to assess agreement between echocardiography and CMR classification, and logistic regression was used to determine univariate predictors of misclassification. A p value of 0.05 was regarded as statistically significant.

## Results

**Figure 1:**
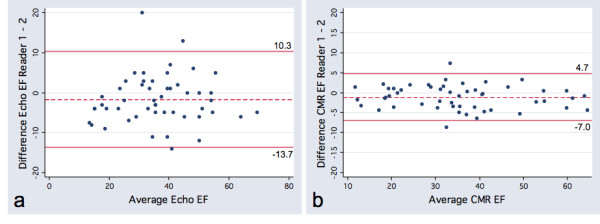
**Bland Altman plots for interobserver variability.a**, Echocardiographic Ejection Fraction (Echo EF) and** b**, Cardiac Magnetic Resonance Ejection Fraction (CMR EF), with limits of agreement.

**Figure 2:**
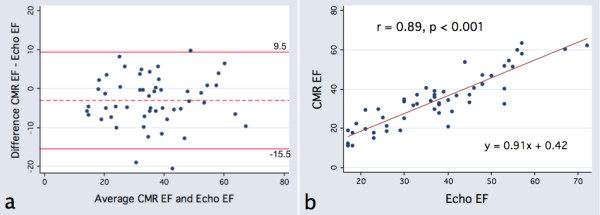
**Comparison of CMR and echocardiographic Ejection Fraction.a**, Bland-Altman plot and **b**, Scatterplot.

Fifty-two (52) patients were identified. The mean age was 62 +/− 15 years and 42 (81%) were male. There was a median of 3 days (interquartile range 1–9) between echocardiographic and CMR studies. (Table 1) CMR was found to have greater reproducibility than echocardiography for both intra-observer (ICC 0.98 vs 0.94) and inter-observer comparisons (ICC 0.99 vs 0.93). The limits of agreement were also substantially narrower for CMR as compared with echocardiography (Figure 1) (Table 2). Using CMR as the reference standard, echocardiography was found to systematically overestimate EF by an average of 3 percentage points (37.5% vs 34.4%, p = 0.001). More importantly, the random error between echocardiography and CMR was substantial, as evidenced as by the limits of agreement (−15.5, 9.5) when comparing techniques (Figure 2). There was no relationship between the time interval between echocardiography and CMR and the absolute difference in EF between the two techniques (r = −0.03, p = 0.85).

**Table 1:** Characteristics of Study Population

	**n = 52**
Age (years) ± SD	62 ± 15
Male Gender	42 (81%)
Ischemic Heart Disease	20 (38%)
Hypertension	21 (40%)
Diabetes mellitus	13 (25%)
Dyslipidemia	37 (78%)
EF by CMR	35 ± 14%
EF by echocardiography	38 ± 14%
LV end-diastolic volume by echocardiography (mL)	165 ± 83
LV end-systolic volume by echocardiography (mL)	111 ± 75
LV end-diastolic volume by CMR (mL)	238 ± 98
LV end-systolic volume by CMR (mL)	166 ± 97
Interval between echocardiography and CMR	3 days (IQR 1 – 9)

**Table 2:** Reproducibility of CMR and Echocardiographic EF

	**CMR**		**Echocardiography**	
	**Intraobserve**r	**Interobserver**	**Intraobserver**	**Interobserver**
ICC (95% CI)	0.98 (0.96 – 0.99)	0.99 (0.98 – 0.99)	0.94 (0.90 – 0.97)	0.93 (0.88 – 0.96)
Pearson’s Correlation Coefficient	0.98	0.98	0.90	0.90
Bland-Altman Bias	1.63	−1.1	1.8	−1.7
Limits of Agreement	−5.3, 8.5	−7.0, 4.7	−10.6, 14.3	−13.7, 10.3

At the EF threshold of 35%, CMR reclassified 11 of 52 (21%) of patients with respect to eligibility for ICD. In 10 of the 11 instances of reclassification, CMR found the EF to be below 35% making the patient potentially ICD-eligible (Table 3). At the EF threshold of 30%, CMR reclassified 5 patients (9.6%) and in all 5 instances determined the patient was ICD-eligible when echocardiography suggested otherwise (Table 4). When the echocardiographic EF was between 25 – 40%, 9 of 22 (41%) of patients were reclassified at either the 30 or 35% EF threshold. On univariate analysis, the only significant predictor of reclassification was an echocardiographic EF within 5 percentage points of the commonly used thresholds (Table 5).

**Table 3:** ICD Eligibility at EF 35% Threshold

		**Echocardiographic EF**	
		**> = 35%**	**< 35%**
CMR EF	>= 35%	21	1
	< 35%	10	20

Kappa = 0.59.

**Table 4:** ICD Eligibility at EF 30% Threshold

		**Echocardiographic EF**	
		**> = 30%**	**< 30%**
CMR EF	>= 30%	32	0
	< 30%	5	15

Kappa = 0.79.

**Table 5:** Predictors of Reclassification of ICD Eligibility by CMR

**Variable**	**Odds Ratio**	**p value**
Days between echocardiography and CMR	0.94 (0.84 – 1.09)	0.30
History of Ischemic Heart Disease	0.64 (0.17 – 2.39)	0.51
Left bundle branch block on electrocardiogram	1.15 (0.26 – 5.16)	0.86
Apical Aneurysm (echocardiography)	0.26 (0.03 – 2.31)	0.23
Regional pattern of left ventricular dysfunction	1.17 (0.32 – 4.26)	0.82
Echocardiographic EF 25 – 40%	6.23 (1.44 – 26.95)	0.01

**Figure 3:**
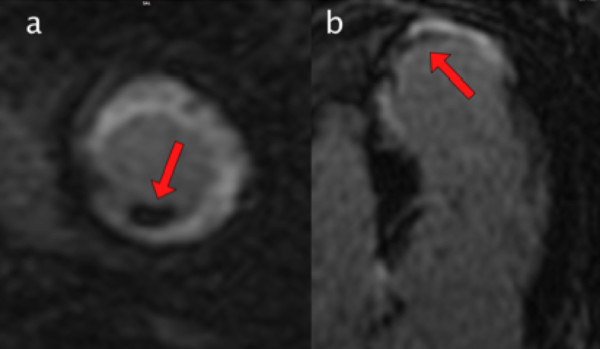
**Gadolinium enhanced CMR viability image.a**. Left ventricular short axis mid-papillary orientation **b**. Apical 4 chamber orientation. Mural thrombus with low signal intensity (arrow) adjacent to blood pool and myocardial scar.

Left ventricular thrombus was identified in one patient by echocardiography and in five additional patients by CMR. The prevalence of unexpected left ventricular thrombus identified by CMR was therefore 9.6% (Figure 3).

## Discussion

Patient selection for ICD implantation is one of the few clinical scenarios in which a precise EF threshold is used. In this study, we found that up to 21% of patients would have been reclassified by CMR regarding ICD eligibility based on EF criteria. Moreover, in those with an echocardiographic EF within 5 percentage points of the commonly used thresholds of 30 or 35%, CMR reclassified 41% of patients, and usually in favour of ICD implantation. Given the clinical importance of ICDs and their expense, the potential impact of CMR in this patient group is substantial.

Several studies have examined EF derived from different imaging modalities in patients being considered for ICD implantation [15-19]. Radionucleotide ventriculography, although not used in this study, is a modality frequently used in patients being considered for ICD implantation [17]. When available however, CMR remains the technique of choice, not only because of it’s greater reproducibility, but because it spares the patient significant radiation exposure and may provide other useful information in heart failure patients [20].

Our findings regarding CMR and echocardiographic EF are in keeping with prior research. CMR has been shown to have less intra and inter-observer variability than echocardiography, and its higher inter-study reproducibility makes it the modality of choice for detecting changes in EF over time [7,9]. The systematic bias between CMR and echocardiographic EF has been variable in published research. [7-9,21,22]. This discrepancy most likely reflects differences in local practice regarding echocardiographic EF measurement and different conventions for CMR EF quantification. For example, in this study papillary muscles were excluded from the left ventricular mass by CMR, a convention that leads to a lower CMR ejection fraction than when papillary muscles are traced and included in the left ventricular myocardial mass [23,24]. On echocardiography, papillary muscles are excluded from the left ventricular mass when measuring Simpson’s biplane EF and therefore the same methodology - the standard in the participating institutions - was employed for CMR. The systematic bias between echocardiographic and CMR EF in this study is however eclipsed by the large random error when comparing techniques. Potential sources of discrepancy between echocardiography and CMR EF were examined in our cohort of patients (Table 5). Although Simpson’s biplane method has limitations in geometrically distorted ventricles, neither a regional pattern of left ventricular dysfunction nor ischemic etiology were predictors of reclassification for ICD eligibility by CMR. Similarly, the presence of left bundle branch block, as a surrogate for dyssynchrony, was not found to a predictor. While our study was underpowered for this purpose, it is likely that the influence of any of these factors would be overshadowed by the proximity of the echocardiographic EF to the ICD eligibility threshold. That is, regardless of other characteristics, patients with echocardiographic EFs close to 30 or 35% are the ones most likely to fall on the other side of the threshold after CMR.

In our cohort of 52 patients, standard 2-dimensional echocardiography identified only 1 of the 6 patients with left ventricular thrombi seen on CMR. These findings are in keeping with the known low sensitivity of echocardiography for left ventricular thrombus detection [13]. Use of echocardiographic contrast agent is known to improve the sensitivity of echocardiography, although layered mural thrombi would be difficult to detect even with contrast [14]. In a general heart failure population, the identification of left ventricular thrombus may not necessarily alter management because the risk of clinical embolization is thought to be low [25]. However, left ventricular thrombus may be of greater clinical importance in ICD patients as defibrillation threshold testing would generally be avoided [26]. Few patients underwent defibrillator threshold testing in this cohort of patients and there were no thromboembolic sequelae making the true clinical impact of identifying thrombus unclear.

The clinical implication of this study is that the technique used to measure EF in patients being considered for ICD therapy is relevant and may alter management. It is not known whether the use of CMR will lead to improved clinical outcomes as CMR was not used in the initial clinical trials of ICD therapy. Nevertheless, the expense associated with ICD implantation makes precise EF measurement for patient selection desirable [16]. Previous research has suggested that if the echocardiographic EF is < 20%, no further testing for EF is necessary, and our findings would support this conclusion [17].

There are several limitations to this study. Importantly, this study assessed the potential rather than actual clinical impact of CMR. Echocardiographic reports did not always quote a specific ejection fraction or grade of left ventricular dysfunction that would allow classification regarding ICD eligibility. Therefore, all echocardiograms were re-read, as were the CMR studies, to obtain reproducibility data and verify the robustness of the CMR ejection fraction measurements. A similar proportion of patients were reclassified by CMR when the original clinical CMR EF data were used. The true clinical impact of CMR was difficult to ascertain due to the variability in practice between treating physicians and other factors, such as ICD implantation being guided by a nuclear medicine EF despite CMR results. With regard to thrombus detection, as there were no thromboembolic sequalae and no repeat CMR scans (due partly to ICD implantation) we do not have other supporting evidence that the findings noted on CMR were indeed thrombus. CMR is however considered highly accurate for thrombus detection and the chronic nature of the left ventricular dysfunction in this study makes the main differential diagnosis, microvascular obstruction, unlikely [12,13]. The findings of this study also may not be generalizable to other centers given the variability in echocardiographic, and to a less extent CMR, measurement techniques. Neither contrast nor 3-dimensional echocardiography were used, both of which have been shown to increase the accuracy of EF assessment [21,22,27,28]. Finally, as mentioned earlier, the lack of outcome data leaves unanswered the question of whether EF measurement by echocardiography or CMR has greater prognostic significance.

## Conclusion

In this cohort of patients being considered for ICD implantation, CMR was substantially more reproducible than echocardiography for EF and the agreement between techniques was only modest. Using strict EF criteria, CMR would have resulted in almost 21% of patients being reclassified regarding ICD eligibility, with 41% being reclassified if the echocardiographic EF was between 25 and 40%. Left ventricular thrombus was also uncovered by CMR in almost 10% of patients. CMR may alter management in a substantial proportion of potential ICD candidates and should be strongly considered in this patient group. Further trials of ICD therapy guided by CMR findings are awaited.

## Competing interests

The authors have no competing interests with regard to this manuscript.

## Authors’ contributions

Original study idea: SBJ; Original Design: SBJ, HLP, AAH, ATY, KAC; Refinement of Design: AK, AMC, GAW; Reporting studies: SBJ, HLP, LJJ, MH, ATY, KAC; Data Analysis: SBJ, ATY; Manuscript preparation: SBJ, HLP, ATY, KAC, AMC, PD, IM; Referring clinicians: PD, IM, AAH. All authors contributed to drafting and reviewing the manuscript, in addition to approving the final manuscript.

## 

This study received no external funding.
